# Association of New Loci Identified in European Genome-Wide Association Studies with Susceptibility to Type 2 Diabetes in the Japanese

**DOI:** 10.1371/journal.pone.0026911

**Published:** 2011-10-26

**Authors:** Toshihiko Ohshige, Minoru Iwata, Shintaro Omori, Yasushi Tanaka, Hiroshi Hirose, Kohei Kaku, Hiroshi Maegawa, Hirotaka Watada, Atsunori Kashiwagi, Ryuzo Kawamori, Kazuyuki Tobe, Takashi Kadowaki, Yusuke Nakamura, Shiro Maeda

**Affiliations:** 1 Laboratory for Endocrinology and Metabolism, RIKEN Center for Genomic Medicine, Yokohama, Kanagawa, Japan; 2 Department of Internal Medicine, Division of Metabolism and Endocrinology, St. Marianna University School of Medicine, Kawasaki, Kanagawa, Japan; 3 First Department of Internal Medicine, Faculty of Medicine, Toyama University, Toyama, Japan; 4 Health Center, Keio University School of Medicine, Tokyo, Japan; 5 Division of Endocrinology and Metabolism, Department of Internal Medicine, Kawasaki Medical School, Kurashiki, Okayama, Japan; 6 Department of Medicine, Shiga University of Medical Science, Otsu, Shiga, Japan; 7 Department of Medicine, Metabolism and Endocrinology, School of Medicine, Juntendo University, Tokyo, Japan; 8 Sportology Center, Graduate School of Medicine, Juntendo University, Tokyo, Japan; 9 Department of Diabetes and Metabolic Diseases, Graduate School of Medicine, The University of Tokyo, Tokyo, Japan; 10 Laboratory of Molecular Medicine, Human Genome Center, Institute of Medical Science, University of Tokyo, Tokyo, Japan; University of Hyderabad, India

## Abstract

**Background:**

Several novel susceptibility loci for type 2 diabetes have been identified through genome-wide association studies (GWAS) for type 2 diabetes or quantitative traits related to glucose metabolism in European populations. To investigate the association of the 13 new European GWAS-derived susceptibility loci with type 2 diabetes in the Japanese population, we conducted a replication study using 3 independent Japanese case-control studies.

**Methodology/Principal Findings:**

We examined the association of single nucleotide polymorphisms (SNPs) within 13 loci (*MTNR1B*, *GCK*, *IRS1*, *PROX1*, *BCL11A*, *ZBED3*, *KLF14*, *TP53INP1*, *KCNQ1*, *CENTD2*, *HMGA2*, *ZFAND6 and PRC1*) with type 2 diabetes using 4,964 participants (2,839 cases and 2,125 controls) from 3 independent Japanese samples. The association of each SNP with type 2 diabetes was analyzed by logistic regression analysis. Further, we performed combined meta-analyses for the 3 studies and previously performed Japanese GWAS data (4,470 cases vs. 3,071 controls). The meta-analysis revealed that rs2943641 in the *IRS1* locus was significantly associated with type 2 diabetes, (P = 0.0034, OR = 1.15 95% confidence interval; 1.05–1.26) and 3 SNPs, rs10930963 in the *MTNR1B* locus, rs972283 in the *KLF14* locus, and rs231362 in the *KCNQ1* locus, had nominal association with type 2 diabetes in the present Japanese samples (P<0.05).

**Conclusions:**

These results indicate that *IRS1* locus may be common locus for type 2 diabetes across different ethnicities.

## Introduction

Type 2 diabetes is a chronic metabolic disorder characterized by hyperglycemia, variable degrees of insulin resistance, and impaired insulin secretion. The total number of individuals with diabetes mellitus is estimated to be nearly 300 million worldwide, and its prevalence continues to increase in many countries, including Japan. Although the precise mechanisms underlying the development and progression of type 2 diabetes have not been elucidated, it is considered that genetic factors play an important role in the pathogenesis of the disease [1]

Currently, approximately 40 susceptibility loci for type 2 diabetes, mostly discovered through genome-wide association studies (GWAS), have been confirmed in populations of European descent (the Wellcome Trust Case Control Consortium/United Kingdom Type 2 Diabetes Genetics consortium [WTCCC/UKT2D], Diabetes Genetics Initiative [DGI], Finland-US Investigation of NIDDM genetics [FUSION], GWAS performed by deCODE genetics, Diabetes Gene Discovery Group [DGDG] and DIAbetes Genetics Replication and Meta-analysis [DIAGRAM]) [2]–[6]. Three Japanese GWAS including ours, have identified the association of the potassium voltage-gated channel KQT-like subfamily member 1 (*KCNQ1*) locus, ubiquitin-conjugating enzyme E2E 2 (*UBE2E2*) locus and C2 calcium-dependent domain containing 4A (*C2CD4A*)-*C2CD4B* locus with type 2 diabetes [7]–[9].

Because many of these loci have also been shown to be associated with type 2 diabetes in other ethnic populations, including Japanese, these loci may be considered convincing susceptibility loci for type 2 diabetes across different ethnicities [10]–[12]. Recently, a locus near insulin receptor substrate 1 (*IRS1*) was identified by GWAS on French patients [13]. Several new loci for type 2 diabetes have been additionally identified through GWAS for quantitative traits related to glucose metabolism, such as fasting plasma glucose (FPG) and 2-hour glucose levels (Meta-Analyses of Glucose and Insulin-related traits Consortium [MAGIC]) [14]–[18]. In addition, 12 novel loci for type 2 diabetes have been identified in an expanded meta-analysis of the existing GWAS data (DIAGRAM+) [19].

In this study, we aim to evaluate the contribution of these new susceptibility loci identified in European GWAS to conferring susceptibility to type 2 diabetes in the Japanese.

## Methods

### Participants and DNA preparation

We selected 4,964 individuals, 2,839 cases and 2,125 controls, from 3 independent Japanese samples.

RIKEN case-control study (1^st^ study): DNA samples were obtained from peripheral blood samples of type 2 diabetes patients recruited from the outpatient clinics of the Shiga University of Medical Science and the Kawasaki Medical School (Case 1; n = 1,630, 978 men and 652 women), We also examined 716 controls who were enrolled in an annual health check conducted either at the Juntendo University or the Keio University (Control 1; n = 716, 465 men and 251 women).

Toyama University study (2^nd^ study): We selected 724 individuals with type 2 diabetes from the outpatient clinic of the Toyama University Hospital (Case 2; n = 724, 451 men and 273 women). We also examined 763 controls with HbA1c<6.0%, age≥50, no family history of diabetes mellitus for the first and second degree relatives (Control 2; n = 763, 359 men and 404 women).

St. Marianna University study (3^rd^ study): We recruited 485 individuals with type 2 diabetes from the outpatient clinic of the St. Marianna University School of Medicine (Case 3; n = 485, 288 men and 197 women). We also examined 646 controls, who were enrolled in an annual health check conducted at the St. Marianna University School of Medicine (Control 3; n = 646, 188 men and 458 women)

The clinical characteristics of the participants are summarized in Table 1. Diabetes was diagnosed according to the World Health Organization (WHO) criteria [20]. Type 2 diabetes is clinically defined as a disease with gradual adult onset. Subjects who tested positive for anti-glutamic acid decarboxylase (GAD) antibodies and those diagnosed to have mitochondrial disease (mitochondrial myopathy, encephalopathy, lactic acidosis, and stroke-like episodes [MELAS]) or maturity onset diabetes of young (MODY) were not included in the case patient groups. Written informed consents were obtained from all participants. DNA was extracted using the standard phenol-chloroform procedure. The protocol was approved by the ethics committees of the RIKEN Yokohama Institute, Shiga University of Medical Science, Keio University, Toyama University, University of Tokyo, Juntendo University and St. Marianna University School of Medicine, or by Institutional Review Board of Kawasaki Medical School.

**Table 1:** Clinical characteristics of the participants.

	RIKEN case-control study1^st^ study		Toyama University study2^nd^ study		St. Marianna University Study3^rd^ study	
	Case	Control	Case	Control	Case	Control
n	1,630	716	724	763	485	646
Sex (M∶F)	978∶652^b^	465∶251	451∶273^b^	359∶404	288∶197^b^	188∶458
Age (year)^a^	61.5±11.6^b^	44.3±9.9	64.9±11.1^b^	72.5±9.0	64.2±11.5^b^	35.0±10.3
BMI (kg^2^/m^2^)^a^	23.7±3.9^b^	22.9±3.1	24.5±3.9^b^	22.7±3.3	24.9±4.6^b^	21.6±3.0
HbA1c (%)^a^	7.79±1.66	N.A.	7.53±1.25^b^	5.54±0.25	7.14±0.99^b^	5.36±0.41
Duration (year)^a^	17.3±8.5	-	13.5±9.1	-	15.9±14.6	-

a Data are mean ± SD. N.A. Not Available.

b P<0.01 vs. Control.

### Single nucleotide polymorphisms genotyping

We first selected 9 single nucleotide polymorphisms (SNPs) from previous reports, rs2943641 within the locus near the *IRS1* [13], and 8 SNPs from the loci identified through GWAS for quantitative traits related to glucose metabolism (rs10830963 [14], [15] and rs1387153 [16] in the melatonin receptor 1B [*MTNR1B*] locus, rs780094 in the glucokinase regulator [*GCKR*] locus, rs730497 in the glucokinase [*GCK*] locus, rs11708067 and rs2877716 in the adenylate cyclase 5 [*ADCY5*] locus, rs2191349 in the diacylglycerol kinase, beta [*DGKB*]- transmembrane protein 195 [*TMEM195*] locus, and rs340874 in the prospero homeobox 1 [*PROX1*] locus [17], [18]). We also examined 11 autosomal SNPs from 11 novel loci for type 2 diabetes recently identified in an expanded meta-analysis of existing GWAS data (rs243021 in the B-cell CLL/lymphoma 11A [*BCL11A*] locus, rs4457053 in the zinc finger, BED-type containing 3 [*ZBED3*] locus, rs972283 in the Kruppel-like factor 14 [*KLF14*] locus, rs896854 in the tumor protein p53 inducible nuclear protein 1 [*TP53INP1*] locus, rs13292136 in the coiled-coil-helix-coiled-coil-helix domain containing 9 [*CHCHD9*] locus, rs231362 in the *KCNQ1* locus, rs1552224 in the centaurin, delta 2 [*CENTD2*] locus, rs1531343 in the high mobility group AT-hook 2 [*HMGA2*] locus, rs7957197 in the 2′-5′-oligoadenylate synthetase-like [*OASL*] locus, rs11634397 in the zinc finger, AN1-type domain 6 [*ZFAND6*] locus, and rs8042680 in the protein regulator of cytokinesis 1 [*PRC1*] locus) [19]. Among them, 2 SNP loci, rs780094 in the *GCKR* locus and rs2191349 in the *DGKB*- *TMEM195* locus were shown to be associated with type 2 diabetes in the Japanese [9], [21], and excluded from the present study.

SNP genotyping was performed by the multiplex-polymerase chain reaction (PCR)-invader assay, as described previously [22].

### Statistical analysis

We performed the Hardy-Weinberg Equilibrium (HWE) test according to the method described by Nielsen et al [23], and SNPs significantly deviated from HWE proportion (P<0.01) in the control groups were excluded from the present analysis. Genotype distribution differences between the case and control groups were analyzed by logistic regression analysis, and quantitative traits analyses were performed by multiple linear regression analysis. To test the additive model of each SNP with or without adjusting sex and log transformed body mass index (BMI), the analysis was performed using StatView software. Combined meta-analysis was performed by using the Mantel-Haenszel procedure with a fixed effect model after testing for heterogeneity. Bonferroni's method was applied for correcting multiple testing errors. The power of sample size for the present study to identify the association of previously reported SNP loci with type 2 diabetes was calculated using “CaTS power calculator for genetic studies” software (http://www.sph.umich.edu/csg/abecasis/CaTS/).

## Results

Among the 18 SNPs, 3 (rs11708067, rs2877716 in *ADCY5* locus, and rs7957197 in *OASL* locus) are monoallelic in the Japanese populations. The genotype distributions of rs13292136 in *CHCHD9* showed significant deviation from the Hardy-Weinberg equilibrium proportion in the control group (P<0.01, Table S1). Therefore, we removed these 4 SNPs from the association study. Then, we examined the association of 14 SNPs within the 13 loci with type 2 diabetes in 3 independent Japanese case-control studies (2,839 cases and 2,125 controls). As shown in Table 2, all 13 loci had the same direction of effect (odds ratio >1.0) with those identified in European studies (P = 0.0001, binomial test). Five SNPs, rs10830963 in the *MTNR1B* locus, rs2943641 in the *IRS1* locus, rs972283 in the *KLF14* locus, rs231362 in the *KCNQ1* locus, and rs11634397 in the *ZFAND6* locus, had nominal association with type 2 diabetes in the present Japanese samples (P<0.05, Table 2 and Table S2), but these associations were not remained significant after Bonferroni's correction. When we combined the present results with those in the previously performed Japanese GWAS data (4,470 cases vs. 3,071 controls), 11 out of 13 loci showed directionally consistent association with those in the European populations (P = 0.01, binomial test), and the association of rs2943641 attained statistically significant levels, whereas rs11634397 in *ZFAND6* were no longer associated with the disease.

**Table 2:** Association of 14 SNPs identified in European GWAS with type 2 diabetes in Japanese populations.

SNP	Gene	Risk Allele^a^	RAF(case/control)			Study 1+2+3			+ Previous Japanese GWAS	
			1^st^ study	2^nd^ study	3^rd^ study		*p* value^c^	OR (95%CI)	*p* value^c^	OR(95%CI)
rs1387153	*MTNR1B*	T	0.406/0.387	0.414/0.410	0.416/0.382	unadjustedadjusted^b^	0.0880.55	1.08(0.99–1.17)1.04 (0.92–1.16)	0.037	1.06 (1.00–1.11)
rs10830963	*MTNR1B*	G	0.426/0.403	0.444/0.433	0.438/0.406	unadjustedadjusted^b^	0.0450.28	1.09 (1.00–1.19)1.06 (0.95–1.19)	0.033	1.06 (1.00–1.11)
rs730497	*GCK*	A	0.181/0.182	0.187/0.185	0.174/0.173	unadjustedadjusted^b^	0.970.57	1.00 (0.90–1.12)0.96 (0.84–1.10)	0.84	1.01 (0.94–1.08)
rs2943641	*IRS1*	C	0.925/0.918	0.926/0.901	0.915/0.903	unadjustedadjusted^b^	0.0130.0091	1.21 (1.04–1.40)1.30 (1.07–1.58)	0.0034	1.15 (1.05–1.26)
rs340874	*PROX1*	G	0.389/0.386	0.397/0.363	0.411/0.382	unadjustedadjusted^b^	0.0590.041	1.09 (0.997–1.18)1.12 (1.00–1.25)	0.19	1.04 (0.98–1.09)
rs243021	*BCL11A*	T	0.695/0.700	0.689/0.692	0.690/0.673	unadjustedadjusted^b^	0.900.98	1.01 (0.92–1.10)0.998 (0.89–1.12)	0.39	0.98 (0.92–1.03)
rs4457053	*ZBED3*	G	0.024/0.016	0.021/0.023	0.023/0.020	unadjustedadjusted^b^	0.270.75	1.18 (0.88–1.58)1.06 (0.73–1.55)	0.77	0.97 (0.81–1.17)
rs972283	*KLF14*	G	0.760/0.720	0.750/0.723	0.735/0.735	unadjustedadjusted^b^	0.00540.052	1.14 (1.04–1.26)1.13 (0.999–1.27)	0.017	1.07 (1.01–1.14)
rs896854	*TP53INP1*	A	0.307/0.317	0.310/0.295	0.321/0.308	unadjustedadjusted^b^	0.700.52	1.02 (0.93–1.11)1.04 (0.92–1.17)	0.43	1.02 (0.97–1.08)
rs231362	*KCNQ1*	C	0.902/0.899	0.921/0.910	0.925/0.890	unadjustedadjusted^b^	0.0250.10	1.18 (1.02–1.36)1.17 (0.97–1.41)	0.009	1.12 (1.03–1.23)
rs1552224	*CENTD2*	T	0.965/0.966	0.964/0.955	0.971/0.963	unadjustedadjusted^b^	0.220.40	1.15 (0.92–1.43)1.13 (0.85–1.49)	0.058	1.14 (0.996–1.31)
rs1531343	*HMGA2*	C	0.137/0.124	0.124/0.141	0.151/0.127	unadjustedadjusted^b^	0.390.61	1.06 (0.93–1.19)0.96 (0.82–1.12)	0.11	1.06 (0.99–1.15)
rs11634397	*ZFAND6*	G	0.129/0.105	0.111/0.114	0.123/0.105	unadjustedadjusted^b^	0.0430.082	1.14 (1.00–1.30)1.16 (0.98–1.38)	0.35	1.04 (0.96–1.13)
rs8042680	*PRC1*	A	0.998/0.995	0.999/0.999	0.997/0.999	unadjustedadjusted^b^	0.330.13	1.49 (0.64–3.48)2.50 (0.76–8.18)	0.28	1.40 (0.74–2.64)

a risk allele reported in the previous reports.

b adjusting sex, age and log-transformed BMI.

c Nominal P values are presented.

We further examined the association of each SNP with glycemic parameters, HOMA-IR, HOMA-β, and FPG using control samples of the 1^st^ and 2^nd^ study (Table 3). In this analysis, the reported type 2 diabetes risk alleles for rs1387153 and rs10830963 in the *MTNR1B* locus had significant association with reduced beta-cell function or increased FPG as reported previously. Further, the risk allele of rs1531343 in the *HMGA2* locus was significantly associated with increased FPG.

**Table 3:** Association of the 14 SNPs with quantitative traits related to glucose metabolism in the 1st and 2nd study controls.

SNP	Gene	Risk allele^a^		HOMA-IR^b^		HOMA-ß^b^		FPG^c^	
				Effect (SE)	*p*	Effect (SE)	*p* value	Effect (SE)	*p*
rs1387153	*MTNR1B*	T	unadjustedadjusted^d^	−0.008 (0.038)−0.022 (0.035)	0.840.53	−6.942 (2.092)−7.071 (1.967)	0.00090.0003	1.471 (0.443)1.308 (0.422)	0.00090.002
rs10830963	*MTNR1B*	G	unadjustedadjusted^d^	−0.051 (0.038)−0.044 (0.034)	0.180.20	−6.957 (2.064)−6.316 (1.944)	0.00080.0012	1.445 (0.441)1.381 (0.420)	0.00110.001
rs730497	*GCK*	A	unadjustedadjusted^d^	−0.024 (0.046)−0.027 (0.042)	0.600.53	−2.020 (2.544)−1.897 (2.386)	0.430.43	0.467 (0.552)0.366 (0.524)	0.400.49
rs2943641	*IRS1*	C	unadjustedadjusted^d^	0.105 (0.061)0.136 (0.055)	0.0840.014	4.141 (3.367)4.760 (3.149)	0.220.13	0.180 (0.749)0.311 (0.711)	0.810.66
rs340874	*PROX1*	G	unadjustedadjusted^d^	−0.067 (0.038)−0.087 (0.035)	0.0780.013	−3.193 (2.089)−2.932 (1.960)	0.130.14	0.640 (0.446)0.416 (0.425)	0.150.33
rs243021	*BCL11A*	T	unadjustedadjusted^d^	0.014 (0.041)0.012 (0.037)	0.730.75	−0.490 (2.238)0.208 (2.106)	0.830.92	0.639 (0.472)0.515 (0.450)	0.180.25
rs4457053	*ZBED3*	G	unadjustedadjusted^d^	−0.074 (0.119)−0.081 (0.107)	0.530.45	−2.313 (6.621)−2.729 (6.181)	0.730.66	0.691 (1.543)0.902 (1.465)	0.650.54
rs972283	*KLF14*	G	unadjustedadjusted^d^	−0.030 (0.042)−0.047 (0.037)	0.470.21	1.269 (2.287)0.492 (2.140)	0.580.82	−0.173 (0.484)−0.290 (0.460)	0.720.53
rs896854	*TP53INP1*	A	unadjustedadjusted^d^	0.003 (0.041)−0.007 (0.37)	0.950.84	0.940 (2.242)0.558 (2.104)	0.680.79	−0.422 (0.474)−0.314 (0.450)	0.370.49
rs231362	*KCNQ1*	C	unadjustedadjusted	−0.057 (0.064)−0.053 (0.058)	0.370.36	−0.092 (3.517)−0.469 (3.300)	0.980.89	−0.305 (0.737)−0.184 (0.701)	0.680.79
rs1552224	*CENTD2*	T	unadjustedadjusted^d^	−0.031 (0.091)0.004 (0.082)	0.730.96	−2.219 (5.035)−0.596 (4.708)	0.660.90	−2.915 (1.103)−2.762 (1.047)	0.00830.0085
rs1531343	*HMGA2*	C	unadjustedadjusted^d^	0.101 (0.052)0.101 (0.047)	0.0530.032	1.855 (2.868)1.336 (2.706)	0.520.62	1.840 (0.612)1.780 (0.585)	0.00270.0024
rs11634397	*ZFAND6*	G	unadjustedadjusted^d^	−0.061 (0.059)−0.050 (0.054)	0.300.35	0.895 (3.283)0.984 (3.076)	0.790.75	0.390 (0.709)0.409 (0.674)	0.580.54
rs8042680	*PRC1*	A	unadjustedadjusted^d^	−0.586 (0.385)−0.733 (0.347)	0.130.035	2.873 (21.24)−7.444 (19.87)	0.890.71	−2.917 (3.703)−2.407 (3.515)	0.430.49

Results of linear regression analyses.

a risk allele for type 2 diabetes reported in the previous reports.

b n = 925,

c n = 1,378.

d adjusting sex , age and log-transformed BMI.

## Discussion

In the present study, we examined 14 SNPs within 13 susceptibility loci for type 2 diabetes in 3 independent Japanese samples, and identified that rs2943641 near *IRS1* was significantly associated with type 2 diabetes when we combined the present data with those in the previous Japanese GWAS data.

GWAS conducted in European and East Asian populations have revealed multiple risk-associated loci for type 2 diabetes, and some of them have been confirmed and shown to be common across different ethnic groups [24].

In this study, we identified a significant association of rs2943641 near *IRS1* locus, with type 2 diabetes in the Japanese population. The risk allele (C) was consistent with that of a previous study in European populations [13], suggesting this locus is a common susceptibility locus for type 2 diabetes across different ethnic groups. We further identified nominal associations of 3 SNPs with type 2 diabetes in the Japanese population. The risk alleles of these SNPs were consistent with those identified in a European study, suggesting that these 3 SNPs were also good candidates for association with type 2 diabetes in the Japanese. Moreover, most of 13 loci showed directionally consistent association with the previous report; therefore, there are several possibilities for the lack of replication. Regarding ethnic differences, there are moderate heterogeneity in effect size for 4 loci, rs730497, rs340874, rs243021 and rs11634397 (50<I^2^<80), whereas no heterogeneity in effect size (odds ratio) was observed on the remaining loci (data not shown). We could not observe remarkable differences in LD structures around each locus between Japanese and European populations (Figure S1). Therefore a lack of replication for most loci might be explained by the insufficient power of the present study (5–63% for the present 3 studies, 5–95% for the 3 studies + previous GWAS samples if we set a cut-off value at P = 0.05 and the prevalence of type 2 diabetes is assumed to be 10%, Table S3).

The analyses of quantitative traits related to glucose metabolism revealed that SNPs in the *MTNR1B* locus were significantly associated with decreased beta-cell function or increased FPG as reported previously (Table 3). In this analysis, we also found that *HMGA2* locus was significantly associated with increased FPG in these samples, suggesting that SNPs in the *MTNR1B* or the *HMGA2* loci confer susceptibility to type 2 diabetes in Japanese populations. In addition, risk allele of rs2943641 near *IRS1* tended to be associated with increase in HOMA-IR as reported previously, further confirming the contribution of this locus with susceptibility to type 2 diabetes in the Japanese.

### Limitations

The present study has some limitations. First, the present sample size is not sufficiently large to detect true associations for some loci as described above. Second, control subjects in the 1^st^ and 3^rd^ study are younger than type 2 diabetes patients. Although, results were not affected by adjusting age, these limitations may increase the possibility for type 2 error.

In conclusion, these results indicate that the *IRS1* locus is considered common locus involved in susceptibility to type 2 diabetes across different ethnic groups. Three loci—*MTNR1B*, *KLF14*, and *KCNQ1* (independent locus from a locus identified in Japanese GWAS)— may also have some effects. Further studies are required to elucidate the association of these as well as other loci with susceptibility to type 2 diabetes, and to understand the biological significance of these genes and their polymorphisms.

## Supporting Information

Figure S1 **Linkage disequilibrium structures for 500 kb region around each SNP locus in JPT and in CEU.** Pairwise correlation structure analyzed by Haploview (http://www.broadinstitute.org/haploview/haploview). The plot includes pairwise D′ values from the HapMap release 27.
(PDF)

Table S1 **Genotype data for 15 SNPs in the 3 independent Japanese samples.**
(DOC)

Table S2 **The associations of the 14 SNPs with type 2 diabetes in 3 independent Japanese samples.**
(DOC)

Table S3 **Power estimation for each SNP locus in the present study.**
(DOC)
