# Supplementary material for: Association of New Loci Identified in European Genome-Wide Association Studies with Susceptibility to Type 2 Diabetes in the Japanese
Source: PLoS One. 2011 Oct 26;6(10):e26911. doi: 10.1371/journal.pone.0026911 (PMC3202571; doi:10.1371/journal.pone.0026911)
Supplement: Figure S1 — Linkage disequilibrium structures for 500 kb region around each SNP locus in JPT and in CEU. Pairwise correlation structure analyzed by Haploview (http://www.broadinstitute.org/haploview/haploview). The plot includes pairwise D′ values from the HapMap release 27. (PDF) [file pone.0026911.s001.pdf]

MTNR1B

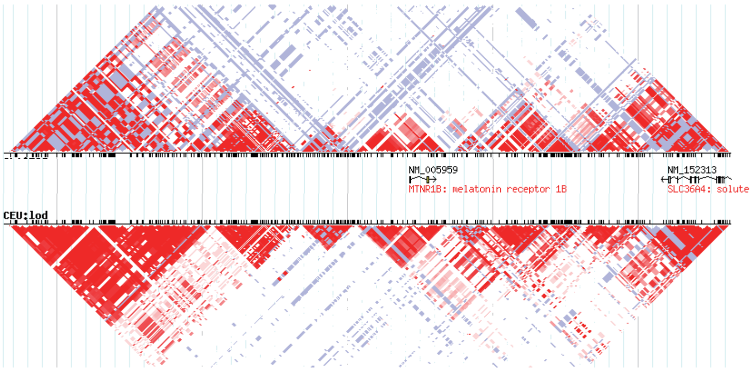

ZBED3

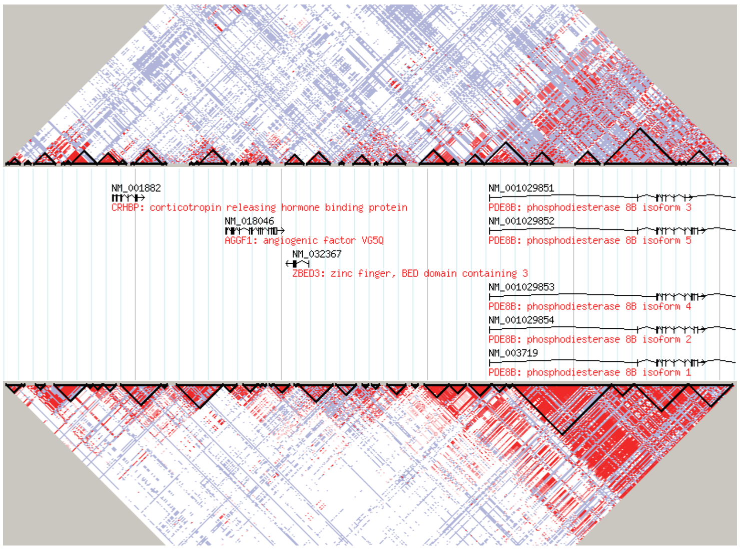

GCK

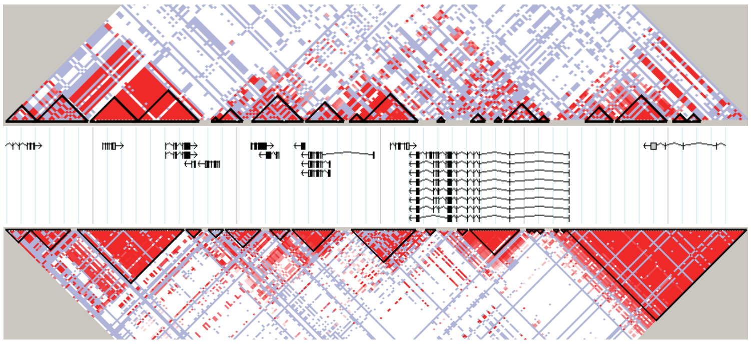

KLF14

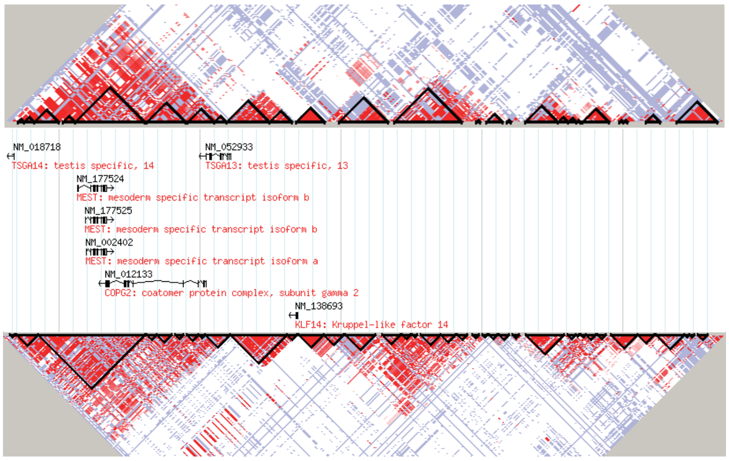

IRS1

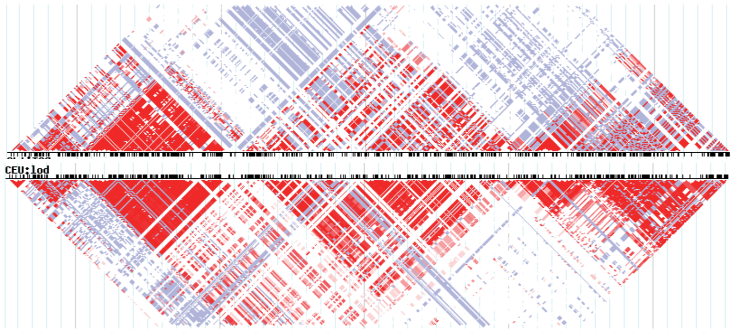

TP53INP1

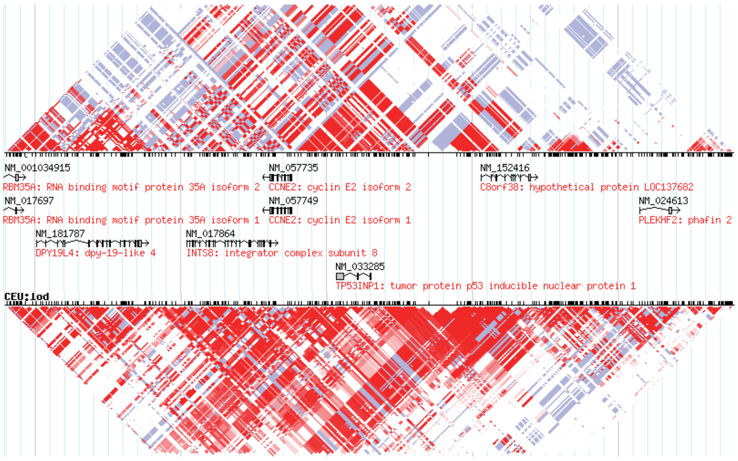

PROX1

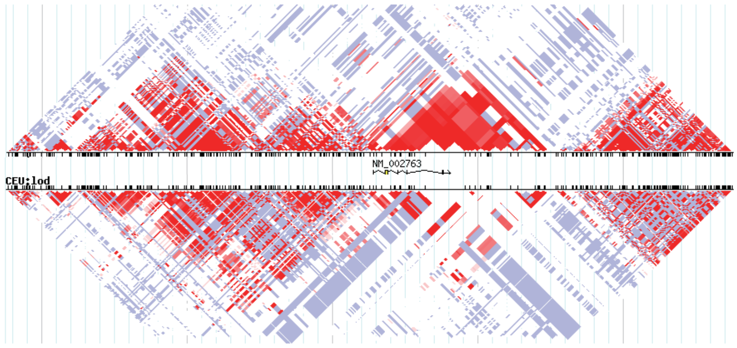

BCL11A

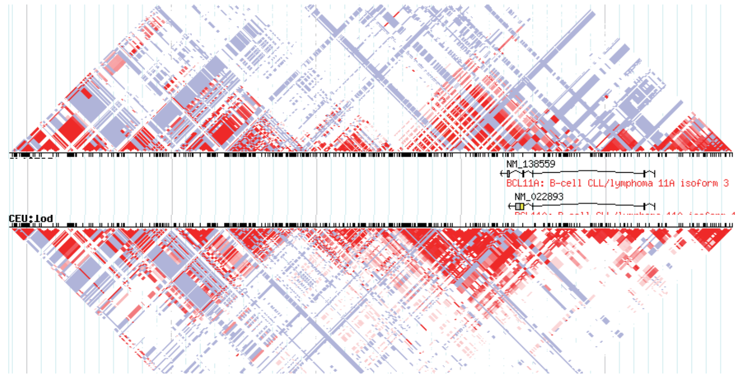

KCNQ1

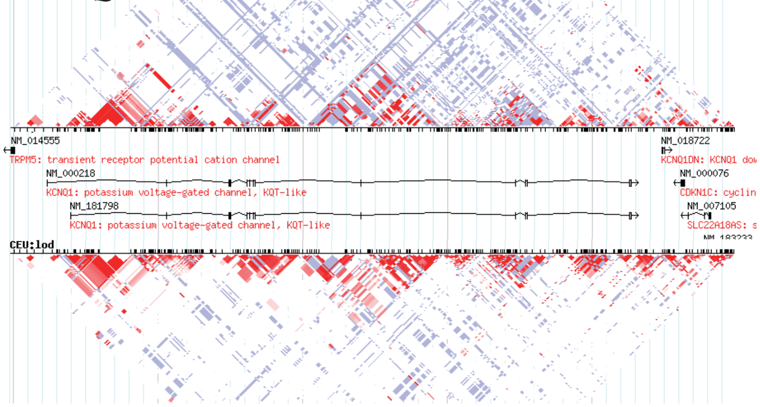

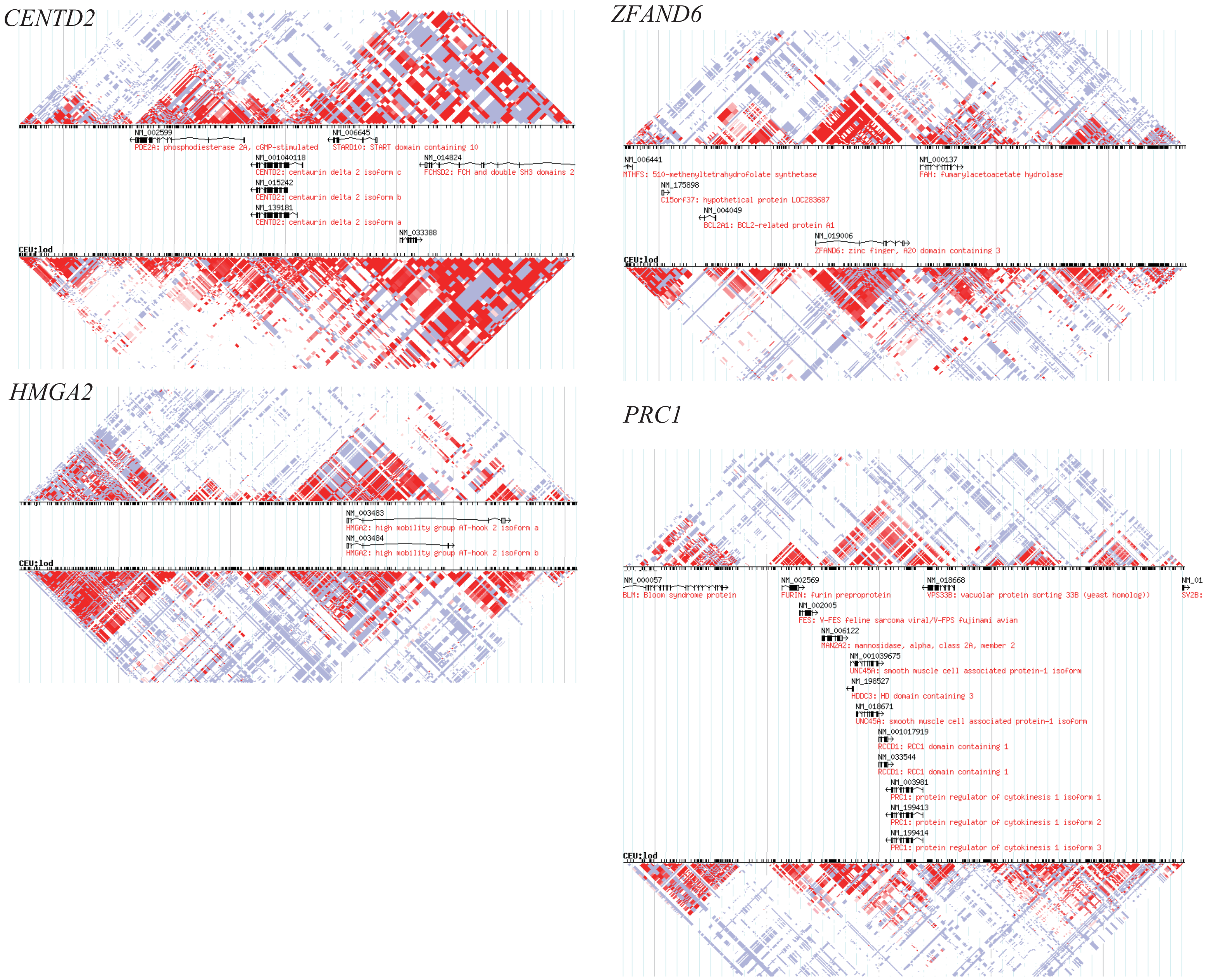

Figure S1 Linkage disequilibrium structures for 500 kb region around each SNP locus in JPT (upper) and in CEU (lower).

Pairwise correlation structure analyzed by Haploview (http://www.broadinstitute.org/haploview/haploview). The plot includes pairwise D’ values from the HapMap release 27.
