# Supplementary material for: Association of New Loci Identified in European Genome-Wide Association Studies with Susceptibility to Type 2 Diabetes in the Japanese
Source: PLoS One. 2011 Oct 26;6(10):e26911. doi: 10.1371/journal.pone.0026911 (PMC3202571; doi:10.1371/journal.pone.0026911)
Supplement: Table S1 — Genotype data for 15 SNPs in the 3 independent Japanese samples. (DOC) [file pone.0026911.s002.doc]

**Table S1** Genotype data for 15 SNPs in the 3 independent Japanese samples

| SNP | Gene | Risk  Allele^a^ | Study | 11/12/22 | | Allele Frequency(1/2) | | P for HWE test | |
| --- | --- | --- | --- | --- | --- | --- | --- | --- | --- |
|  |  |  |  | Case | Control | Case | Control | Case | Control |
| rs1387153 | *MTNR1B* | T  Allele2 | 1^st^  2^nd^  3^rd^  SUM | 561/773/262  249/344/125  169/212/90  979/1329/477 | 253/312/102  253/387/117  246/299/95  752/998/314 | 0.594/0.406  0.586/0.414  0.584/0.416  0.590/0.410 | 0.613/0.387  0.590/0.410  0.618/0.382  0.606/0.394 | 0.47 | 0.57 |
| rs10830963 | *MTNR1B* | G  Allele2 | 1^st^  2^nd^  3^rd^  SUM | 537/776/299  227/344/146  163/215/103  927/1335/548 | 237/327/107  235/387/134  221/317/101  693/1031/342 | 0.574/0.426  0.556/0.444  0.562/0.438  0.567/0.433 | 0.597/0.403  0.567/0.433  0.594/0.406  0.585/0.415 | 0.088 | 0.21 |
| rs730497 | *GCK* | A  Allele2 | 1^st^  2^nd^  3^rd^  SUM | 1083/487/49  487/200/35  323/140/13  1893/827/97 | 449/201/22  502/229/25  435/183/19  1386/613/66 | 0.819/0.181  0.813/0.187  0.826/0.174  0.819/0.181 | 0.818/0.182  0.815/0.185  0.827/0.173  0.820/0.180 | 0.57 | 0.86 |
| rs2943641 | *IRS1* | C  Allele1 | 1^st^  2^nd^  3^rd^  SUM | 1369/227/6  619/101/3  403/76/3  2391/404/12 | 545/101/3  620/131/10  521/114/5  1686/346/18 | 0.925/0.075  0.926/0.074  0.915/0.085  0.924/0.076 | 0.918/0.082  0.901/0.099  0.903/0.097  0.907/0.093 | 0.25 | 0.96 |
| rs340874 | *PROX1* | G  Allele2 | 1^st^  2^nd^  3^rd^  SUM | 590/770/237  266/332/118  166/236/80  1022/1338/435 | 253/310/102  300/350/96  252/290/100  805/950/298 | 0.611/0.389  0.603/0.397  0.589/0.411  0.605/0.395 | 0.614/0.386  0.637/0.363  0.618/0.382  0.623/0.377 | 0.93 | 0.51 |
| rs243021 | *BCL11A* | T  Allele2 | 1^st^  2^nd^  3^rd^  SUM | 152/673/776  79/290/351  47/205/231  278/1168/1358 | 65/269/330  64/331/351  61/290/279  190/890/960 | 0.305/0.695  0.311/0.689  0.310/0.690  0.307/0.693 | 0.300/0.700  0.308/0.692  0.327/0.673  0.311/0.689 | 0.25 | 0.43 |
| rs4457053 | *ZBED3* | G  Allele2 | 1^st^  2^nd^  3^rd^  SUM | 1484/76/0  690/30/0  461/22/0  2635/128/0 | 615/20/0  725/33/1  614/26/0  1954/79/1 | 0.976/0.024  0.979/0.021  0.977/0.023  0.977/0.023 | 0.984/0.016  0.977/0.023  0.980/0.020  0.980/0.020 | 0.21 | 0.83 |
| rs972283 | *KLF14* | G  Allele2 | 1^st^  2^nd^  3^rd^  SUM | 95/570/920  45/266/401  38/179/265  178/1015/1586 | 52/265/342  55/304/388  45/251/348  152/820/1078 | 0.240/0.760  0.250/0.750  0.265/0.735  0.247/0.753 | 0.280/0.720  0.277/0.723  0.265/0.735  0.274/0.726 | 0.36 | 0.82 |
| rs896854 | *TP53INP1* | A  Allele1 | 1^st^  2^nd^  3^rd^  SUM | 146/678/757  75/296/348  38/233/211  259/1207/1316 | 67/285/308  58/333/370  53/281/295  178/899/973 | 0.307/0.693  0.310/0.690  0.321/0.679  0.310/0.690 | 0.317/0.683  0.295/0.705  0.308/0.692  0.306/0.694 | 0.46 | 0.14 |
| rs13292136 | *CHCHD9* | C  Allele1 | 1^st^  2^nd^  3^rd^  SUM | 1300/249/30  598/114/8  381/80/12  2279/443/50 | 533/124/10  623/122/10  499/122/18  1655/368/38 | 0.902/0.098  0.910/0.090  0.890/0.110  0.902/0.098 | 0.892/0.108  0.906/0.094  0.876/0.124  0.892/0.108 | 4.8×10^-7^ | 1.2×10^-3^ |
| rs231362 | *KCNQ1* | C  Allele1 | 1^st^  2^nd^  3^rd^  SUM | 1292/294/9  606/102/5  412/64/4  2310/460/18 | 536/118/8  619/123/6  511/114/13  1666/355/27 | 0.902/0.098  0.921/0.079  0.925/0.075  0.911/0.089 | 0.899/0.101  0.910/0.090  0.890/0.110  0.900/0.100 | 0.34 | 0.11 |
| rs1552224 | *CENTD2* | T  Allele2 | 1^st^  2^nd^  3^rd^  SUM | 2/107/1491  1/50/672  0/28/456  3/185/2619 | 0/45/617  1/67/695  1/46/599  2/158/1911 | 0.035/0.965  0.036/0.964  0.029/0.971  0.034/0.966 | 0.034/0.966  0.045/0.955  0.037/0.963  0.039/0.961 | 0.89 | 0.49 |
| rs1531343 | *HMGA2* | C  Allele1 | 1^st^  2^nd^  3^rd^  SUM | 25/388/1181  4/171/544  15/115/351  44/674/2076 | 17/131/519  21/172/567  8/148/489  46/451/1575 | 0.137/0.863  0.124/0.876  0.151/0.849  0.136/0.864 | 0.124/0.876  0.141/0.859  0.127/0.873  0.131/0.869 | 0.20 | 0.044 |
| rs11634397 | *ZFAND6* | G  Allele2 | 1^st^  2^nd^  3^rd^  SUM | 1222/359/28  567/149/6  377/97/11  2166/605/45 | 532/132/4  592/159/7  519/115/10  1643/406/21 | 0.871/0.129  0.889/0.111  0.877/0.123  0.877/0.123 | 0.895/0.105  0.886/0.114  0.895/0.105  0.892/0.108 | 0.71 | 0.46 |
| rs8042680 | *PRC1* | A  Allele1 | 1^st^  2^nd^  3^rd^  SUM | 1613/7/0  721/1/0  481/3/0  2815/11/0 | 660/7/0  759/2/0  645/1/0  2064/10/0 | 0.998/0.002  0.999/0.001  0.997/0.003  0.998/0.002 | 0.995/0.005  0.999/0.001  0.999/0.001  0.998/0.002 | 0.92 | 0.91 |

^a^ risk allele reported in the previous reports

HWE, Hardy-Weinberg equilibrium
