# Supplementary material for: Association of New Loci Identified in European Genome-Wide Association Studies with Susceptibility to Type 2 Diabetes in the Japanese
Source: PLoS One. 2011 Oct 26;6(10):e26911. doi: 10.1371/journal.pone.0026911 (PMC3202571; doi:10.1371/journal.pone.0026911)
Supplement: Table S2 — The associations of the 14 SNPs with type 2 diabetes in 3 independent Japanese samples. (DOC) [file pone.0026911.s003.doc]

**Table S2** The associations of the 14 SNPs with type 2 diabetes in 3 independent Japanese samples.

| SNP | Gene | Risk  allele^a^ | study | Unadjusted | | Adjusted^b^ | |
| --- | --- | --- | --- | --- | --- | --- | --- |
|  |  |  |  | *p* value | OR (95% CI) | *p* value | OR (95% CI) |
| rs1387153 | *MTNR1B* | T | 1^st^  2^nd^  3^rd^  heterogeneity P  I^2^ | 0.22  0.85  0.11  0.56  0 | 1.09 (0.95-1.24)  1.02 (0.88-1.18)  1.15 (0.97-1.36) | 0.95  0.84  0.19  0.50  0 | 1.01 (0.85-1.19)  1.02 (0.87-1.19)  1.26 (0.89-1.80) |
| rs10830963 | *MTNR1B* | G | 1^st^  2^nd^  3^rd^  heterogeneity P  I^2^ | 0.15  0.57  0.14  0.75  0 | 1.10 (0.97-1.25)  1.04 (0.90-1.21)  1.14 (0.96-1.34) | 0.83  0.59  0.068  0.29  19.7 | 1.02 (0.86-1.21)  1.04 (0.90-1.21)  1.39 (0.98-1.98) |
| rs730497 | *GCK* | A | 1^st^  2^nd^  3^rd^  heterogeneity P  I^2^ | 0.90  0.87  0.86  0.98  0 | 0.99 (0.84-1.17)  1.02 (0.85-1.22)  1.01 (0.81-1.26) | 0.76  0.78  0.56  0.91  0 | 0.97 (0.78-1.20)  0.97 (0.81-1.18)  0.87 (0.54-1.39) |
| rs2943641 | *IRS1* | C | 1^st^  2^nd^  3^rd^  heterogeneity P  I^2^ | 0.36  0.016  0.33  0.49  0 | 1.12 (0.88-1.42)  1.37 (1.06-1.78)  1.16 (0.86-1.55) | 0.43  0.028  0.093  0.47  0 | 1.14 (0.83-1.57)  1.35 (1.03-1.77)  1.72 (0.91-3.24) |
| rs340874 | *PROX1* | G | 1^st^  2^nd^  3^rd^  heterogeneity P  I^2^ | 0.85  0.064  0.17  0.40  0 | 1.01 (0.89-1.16)  1.15 (0.99-1.34)  1.13 (0.95-1.34) | 0.32  0.088  0.50  0.82  0 | 1.09 (0.92-1.30)  1.14 (0.98-1.34)  1.13 (0.80-1.58) |
| rs243021 | *BCL11A* | T | 1^st^  2^nd^  3^rd^  heterogeneity P  I^2^ | 0.76  0.84  0.38  0.64  0 | 0.98 (0.85-1.12)  0.98 (0.84-1.15)  1.09 (0.91-1.30) | 0.95  0.76  0.62  0.84  0 | 1.01 (0.84-1.21)  0.98 (0.83-1.15)  1.10 (0.76-1.59) |
| rs4457053 | *ZBED3* | G | 1^st^  2^nd^  3^rd^  heterogeneity P  I^2^ | 0.076  0.68  0.69  0.30  17.8 | 1.58 (0.95-2.60)  0.90 (0.55-1.47)  1.13 (0.63-2.01) | 0.23  0.63  0.83  0.45  0 | 1.46 (0.79-2.71)  0.88 (0.53-1.47)  0.86 (0.22-3.40) |
| rs972283 | *KLF14* | G | 1^st^  2^nd^  3^rd^  heterogeneity P  I^2^ | 0.005  0.096  0.99  0.17  43 | 1.23 (1.07-1.42)  1.15 (0.98-1.36)  1.00 (0.83-1.21) | 0.088  0.17  0.74  0.57  0 | 1.18 (0.98-1.43)  1.13 (0.95-1.34)  0.94 (0.64-1.38) |
| rs896854 | *TP53INP1* | A | 1^st^  2^nd^  3^rd^  heterogeneity P  I^2^ | 0.48  0.37  0.50  0.45  0 | 0.95 (0.83-1.09)  1.08 (0.92-1.26)  1.07 (0.88-1.29) | 0.63  0.41  0.22  0.37  0.57 | 0.96 (0.80-1.15)  1.07 (0.91-1.26)  1.28 (0.87-1.89) |
| rs231362 | *KCNQ1* | C | 1^st^  2^nd^  3^rd^  heterogeneity P  I^2^ | 0.72  0.26  0.0079  0.16  45.5 | 1.04 (0.84-1.29)  1.16 (0.90-1.51)  1.48 (1.11-1.98) | 0.25  0.30  0.55  0.99  0 | 1.18 (0.89-1.57)  1.15 (0.88-1.51)  1.20 (0.66-2.16) |
| rs1552224 | *CENTD2* | T | 1^st^  2^nd^  3^rd^  heterogeneity P  I^2^ | 0.91  0.20  0.28  0.51  0 | 0.98 (0.69-1.40)  1.27 (0.88-1.84)  1.30 (0.81-2.09) | 0.56  0.18  0.48  0.38  0 | 0.87 (0.55-1.38)  1.30 (0.89-1.91)  1.38 (0.57-3.33) |
| rs1531343 | *HMGA2* | C | 1^st^  2^nd^  3^rd^  heterogeneity P  I^2^ | 0.22  0.19  0.11  0.08  60.4 | 1.13 (0.93-1.37)  0.87 (0.70-1.07)  1.22 (0.96-1.55) | 0.85  0.19  0.38  0.32  12.3 | 1.02 (0.80-1.31)  0.86 (0.69-1.08)  1.24 (0.77-2.02) |
| rs11634397 | *ZFAND6* | G | 1^st^  2^nd^  3^rd^  heterogeneity P  I^2^ | 0.023  0.82  0.20  0.24  30.6 | 1.27 (1.03-1.55)  0.97 (0.77-1.23)  1.18 (0.92-1.53) | 0.0015  0.68  0.92  0.03  72.3 | 1.55 (1.18-2.02)  0.95 (0.75-1.21)  1.03 (0.63-1.69) |
| rs8042680 | *PRC1* | A | 1^st^  2^nd^  3^rd^  heterogeneity P  I^2^ | 0.096  0.60  0.23  0.20  38.1 | 2.44 (0.85-7.00)  1.90 (0.17-21.0)  0.25 (0.03-2.40) | 0.10  0.80  0.75  0.75  0 | 3.14 (0.80-12.3)  1.37 (0.12-16.4)  0.20(0.000009-4230) |

^a^ risk allele for type 2 diabetes reported in the previous reports

^b^ adjusting age, sex and log-transformed BMI
