# Supplementary material for: Association of New Loci Identified in European Genome-Wide Association Studies with Susceptibility to Type 2 Diabetes in the Japanese
Source: PLoS One. 2011 Oct 26;6(10):e26911. doi: 10.1371/journal.pone.0026911 (PMC3202571; doi:10.1371/journal.pone.0026911)
Supplement: Table S3 — Power estimation for each SNP locus in the present study. (DOC) [file pone.0026911.s004.doc]

**Table S3** Power estimation for each SNP locus in the present study

| SNP | Gene | Risk  allele^a^ | RAF in HapMap | | RAF  In Japanese | | Reported OR | Study 1+2+3 | +  GWAS data | Number of samples for 80% power |
| --- | --- | --- | --- | --- | --- | --- | --- | --- | --- | --- |
|  |  |  | CEU | JPT |  |  |  |  |  |  |
| rs1387153 | *MTNR1B* | T | 0.283 | 0.522 | | 0.394 | 1.08^b^ | 52% | 89% | 9,579 |
| rs10830963 | *MTNR1B* | G | 0.300 | 0.489 | | 0.415 | 1.09^c^ | 62% | 95% | 7,495 |
| rs730497 | *GCK* | A | 0.195 | 0.193 | | 0.180 | 1.07^c^ | 30% | 62% | 18,524 |
| rs2943641 | *IRS1* | C | 0.608 | 0.932 | | 0.907 | 1.19^d^ | 63% | 95% | 7,569 |
| rs340874 | *PROX1* | G | 0.508 | 0.330 | | 0.377 | 1.07^c^ | 42% | 79% | 12,315 |
| rs243021 | *BCL11A* | T | 0.458 | 0.739 | | 0.689 | 1.08^b^ | 45% | 83% | 11,403 |
| rs4457053 | *ZBED3* | G | 0.259 | 0.023 | | 0.020 | 1.07^b^ | 8% | 13% | > 20,000 |
| rs972283 | *KLF14* | G | 0.550 | 0.711 | | 0.726 | 1.06^b^ | 27% | 57% | > 20,000 |
| rs896854 | *TP53INP1* | A | 0.475 | 0.284 | | 0.306 | 1.05^b^ | 23% | 49% | > 20,000 |
| rs13292136 | *CHCHD9* | C | 0.933 | 0.867 | | 0.892 | 1.08^b^ | 22% | 48% | > 20,000 |
| rs231362 | *KCNQ1* | C | 0.518 | 0.869 | | 0.900 | 1.07^b^ | 18% | 37% | > 20,000 |
| rs1552224 | *CENTD2* | T | 0.875 | 0.943 | | 0.961 | 1.14^b^ | 22% | 47% | > 20,000 |
| rs1531343 | *HMGA2* | C | 0.100 | 0.144 | | 0.131 | 1.08^b^ | 30% | 62% | 19,089 |
| ßrs11634397 | *ZFAND6* | G | 0.595 | 0.093 | | 0.108 | 1.05^b^ | 13% | 26% | > 20,000 |
| rs8042680 | *PRC1* | A | 0.217 | 1.000 | | 0.999 | 1.06^b^ | 5% | 5% | > 20,000 |

CaTS power calculator, CaTS: <http://www.sph.umich.edu/csg/abecasis/CaTS/>)

The prevalence of type 2 diabetes is assumed to be 10%,  = 0.05

^a^ risk allele for type 2 diabetes reported in the previous reports

^b^ reported by Voight BF et al. (stage 2)

^c^ reported by Dupuis J et al.

^d^ reported by Johan R et al.
